# Identification and Characterization of Phosphoproteins in Somatic Embryogenesis Acquisition during Oil Palm Tissue Culture

**DOI:** 10.3390/plants9010036

**Published:** 2019-12-25

**Authors:** Suvichark Aroonluk, Sittiruk Roytrakul, Chatchawan Jantasuriyarat

**Affiliations:** 1 Department of Genetics, Faculty of Science, Kasetsart University, Bangkok 10900, Thailand; suvichark.a@gmail.com; 2 National Center for Genetic Engineering and Biotechnology (BIOTEC), Klong Luang, Pathumthani 12120, Thailand; sittiruk2000@gmail.com; 3 Center for Advanced Studies in Tropical Natural Resources, National Research University-Kasetsart (CASTNAR, NRU-KU), Kasetsart University, Bangkok 10900, Thailand; 4 Omics Center for Agriculture, Bioresources, Food and Health, Kasetsart University (OmiKU), Kasetsart University, Bangkok 10900, Thailand

**Keywords:** micropropagation, *Elaeis guineensis*, proteomics, nano-liquid chromatography–tandem mass spectrometry

## Abstract

Somatic embryogenesis during oil palm tissue culture is a long process. The identification of the proteins that control this process may help to shorten the time of oil palm tissue culture. We collected embryogenic callus and somatic embryos at the globular, torpedo, and cotyledon maturation stages, as well as from plantlets, for total protein extraction. An enrichment column was used to enrich the phosphoproteins, which were subjected to tryptic enzyme digestion. Each sample was analyzed with nano-liquid chromatography–tandem mass spectrometry (nano LC-MS/MS). A total of 460 phosphoproteins were identified and analyzed. The functional characterization of phosphoproteins were observed as highest in the metabolic process, protein/nucleotide/ion binding, and membrane component. The different phosphoproteins are involved in the control of vegetative growth, cellular differentiation, cell morphogenesis, and signaling roles in plants. The Quantitative Real-Time Reverse Transcription-PCR technique (qPCR) was successfully used to verify the expression of genes, and the results were consistent with the level of protein expression from nano-LC-MS/MS. The E3 ubiquitin-protein ligase and sister chromatid cohesion PDS5 were specifically expressed only in the somatic embryo and plantlet, and these could be used as protein biomarkers to determine the oil palm somatic embryo maturation stage. This study sheds light on the protein phosphorylation mechanism that regulates somatic embryogenesis transition during oil palm tissue culture.

## 1. Introduction

Oil palm (*Elaeis guineensis* Jacq.) belongs to the Arecaceae family and is the largest source of edible vegetable oil worldwide [1]. Oil palm is the most efficient oil-bearing crop, with an average annual yield of 4–5 tons of crude oil per hectare per year [2]. It is useful for food production, non-food derivatives, oleochemicals, and in the biofuel industries. In the last decade, the demand for palm oil has increased rapidly, but the world production of palm oil is far from sufficient to meet the demand. In order to meet the world’s high demand of palm oil, oil palm plantation areas in many oil palm production countries including Malaysia, Indonesia and Thailand are expanding [3]. To comply with this plantation expansion, hundreds of thousands of high-yield oil palm plants will need to be seeded to meet the increased demand. Unfortunately, the propagation by seed of a common commercial oil palm type, Tenera, which is a hybrid between the Dura and Pisifera types, will result in yield segregation and will not meet the true-to-type nature of elite high-yield oil Tenera hybrids [4]. Therefore, a tissue culture was used to produce true-to-type seedlings in the large-scale clonal propagation of elite Tenera [5]. To produce an elite oil palm tree, explants were inoculated on a culture medium supplemented with plant growth regulators, and plantlets were generated by the indirect somatic embryogenesis process. The oil palm tissue culture takes at least 12 months from explant to completely regenerate the plantlet [5,6].

Somatic embryogenesis is a process by which the somatic cell undergoes dedifferentiation or re-differentiation to enter the specific biological programming, giving rise to somatic embryos during clonal micro-propagation. Indirect somatic embryogenesis (ISE) is composed of three differential steps. Step one is the transition from non-embryogenic callus to an embryogenic callus. Step two is the development of the somatic embryo, including the globular stage, the torpedo stage and the cotyledonary stage. The last step is the maturation of the somatic embryo to a plantlet [7]. Karami et al. [8] identified several genes that are involved in the transition process from a somatic cell to an embryogenic cell in plant. Various genes were reported to be involved in somatic embryogenesis in higher plants including the *Leafy cotyledon* gene (*LEC*) by Ledwon et al. [9] and the *Baby Boom* gene (*BBM*) by Kulinskas-Lukaszek et al. [10]. 

In the last decade, somatic embryogenesis has been studied by using proteomic techniques. Takáč et al. [11] reported several oxidative stress proteins involved in somatic embryogenesis, including ascorbate peroxidase (APX) and manganese superoxide dismutase (MnSOD) in grape wine somatic embryo development. Furthermore, Silva Rde et al. [12] used the proteomic technique to identify differentially expressed proteins during the initial stage of oil palm embryogenesis. The results showed that several proteins function in pro-embryogenic callus formation, such as type IIIa membrane protein cp-wap13, fructokinase and PR proteins, which can be developed to biomarkers. However, the study of protein post-translational modification is still elusive. 

Protein phosphorylation is one of the major mechanisms in the post-translational modification (PTM) process. It regulates protein activity to a signal transduction pathway. One-third of all proteins are reversibly phosphorylated in a specific site of the protein structure, which leads to a defined function in enzyme activity, intracellular localization, and protein stability [13,14]. Many proteins can be modified by different kinds of protein kinases that regulate multiple processes including biological metabolism and hormone response [15]. In *Arabidopsis*, phosphoproteins have been reported to account for 4% of all proteins. The phosphorylation and dephosphorylation events generally occur on the serine (Ser), threonine (Thr) and tyrosine (Tyr) amino acid residues [16]. Previously, researchers had studied phosphoproteomics in the moss *Physcomitrella patens* (Hedw.), showing phosphoproteins including somatic embryogenesis receptor kinase and the N-acetyl-L-cysteine (NAC) transcription factor that have a major role in the developmental reprograming of a protoplast [17]. Moreover, the study of wheat callus phosphoproteomic during *in vivo* orthophosphate uptake showed a differential spot of proteins involved in protein synthesis, stress, and reactive oxygen species (ROS) detoxification [18]. However, no one has examined phosphoproteins, which are expressed during oil palm somatic embryogenesis. This study aimed to identify phosphoproteins, which may have important roles in the biological mechanism during the progressive transition of the embryogenic callus to somatic embryogenesis, during oil palm tissue culture.

## 2. Results

### 2.1. Phosphoprotein Enrichment and LC-MS/MS Identification

**Figure 1:**
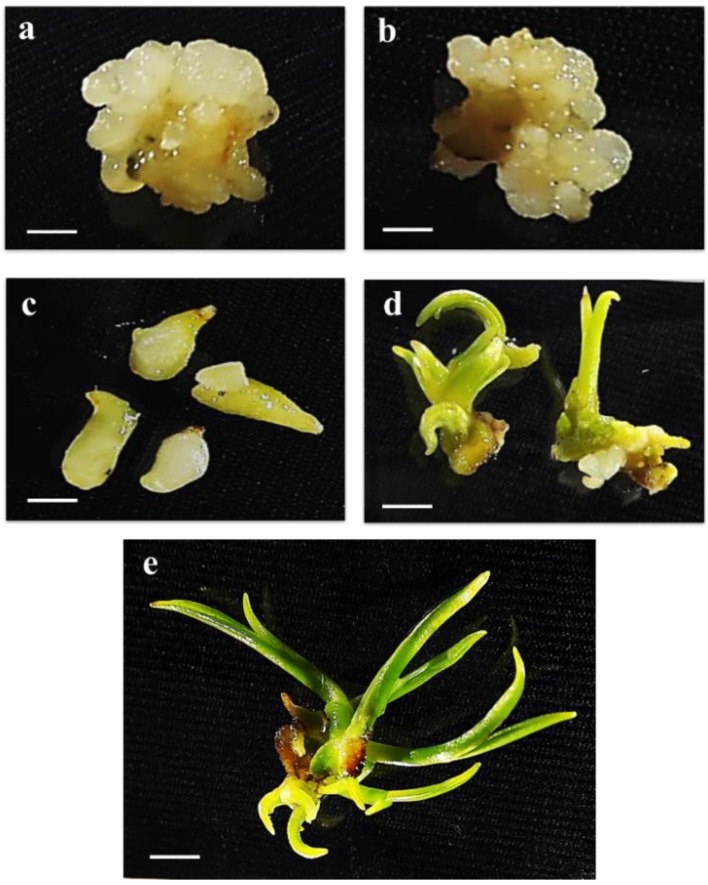
*Elaeis guineensis* Jacq cv “Tenera” developmental stages of somatic embryogenesis. (**a**) embryogenic callus (bar = 0.25 cm), (**b**) globular (bar = 0.25 cm), (**c**) torpedo (bar = 0.25 cm) (**d**) cotyledonary (bar = 0.25 cm), and (**e**) plantlet (bar = 0.25 cm) stages.

**Figure 2:**
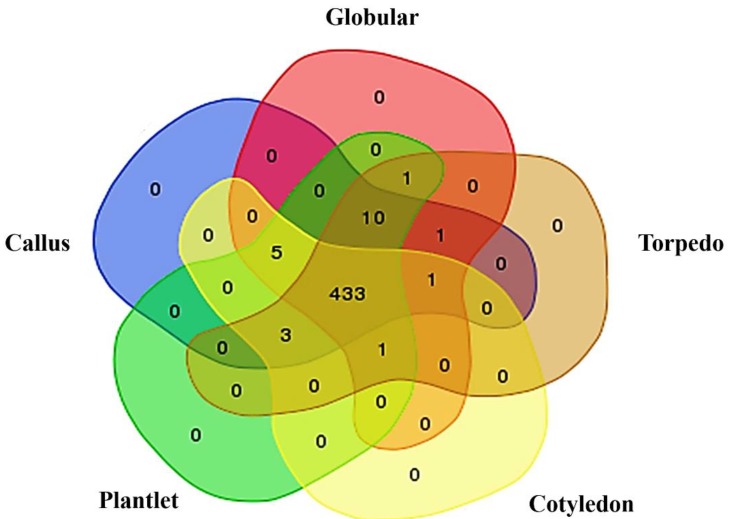
Overlap of identified phosphoproteins in callus (blue), globular (pink), torpedo (brown), cotyledon (yellow) and plantlet (green) stages.

To evaluate the phosphoprotein change during indirect oil palm somatic embryogenesis (Figure 1), peptide sequences from nano-liquid chromatography–tandem mass spectrometry (nano LC-MS/MS) were searched against the *Elaeis guineensis* genome database. From a total of 460 predicted proteins, amounts of 458, 457, 455 and 448 were presented in the callus, globular, torpedo and cotyledonary stages, respectively. Four hundred and thirty-three proteins were shared in all stages. Ten proteins were specifically presented in the callus, globular, torpedo and plantlet stages but not in the cotyledonary stage, namely the uncharacterized protein LOC105047683 isoform X3, pentatricopeptide repeat-containing protein At4g39530, zinc-finger homeodomain protein 6-like, uncharacterized protein LOC105048836 isoform X1, endoribonuclease Dicer homolog 1 isoform X1, cationic amino acid transporter 1-like, AT-rich interactive domain-containing protein 4-like, DNA polymerase eta isoform X1, probable UMP/CMP kinase 4, and uncharacterized protein LOC105047412. Five proteins were specifically expressed in the callus, globular, cotyledon and plantlet stages but not in the torpedo stage, namely myosin-12, acyl carrier protein 2, mitochondrial isoform X2, uncharacterized protein At1g01500, QWRF motif-containing protein 8-like isoform X1 and serine/threonine-protein kinase ULK4. Three proteins were specifically expressed in callus, torpedo, cotyledon and plantlet but not in the globular stage, namely thioredoxin-like 1-2, chloroplastic, uncharacterized protein LOC105042379 and uncharacterized protein LOC105032065. Pollen-specific leucine-rich repeat extensin-like protein 2 was expressed only in the callus, globular, torpedo and cotyledon stages. The E3 ubiquitin-protein ligase At1g63170-like isoform X2 was expressed only in the globular, torpedo, cotyledon and plantlet stages. Probable inactive receptor kinase At1g48480 was specifically expressed in the callus, globular and torpedo stages. The sister chromatid cohesion protein PDS5 homolog A isoform X2 was specifically expressed in the globular, torpedo and plantlet stages (Figure 2). Groups of expressed proteins that were specifically expressed from embryogenic callus acquisition to somatic embryo and somatic embryo differentiation, as seen in the Venn diagram (Figure 2), were collected for verification by determining the mRNA expression levels.

### 2.2. Functional Classification of Identified Phosphoproteins 

**Figure 3:**
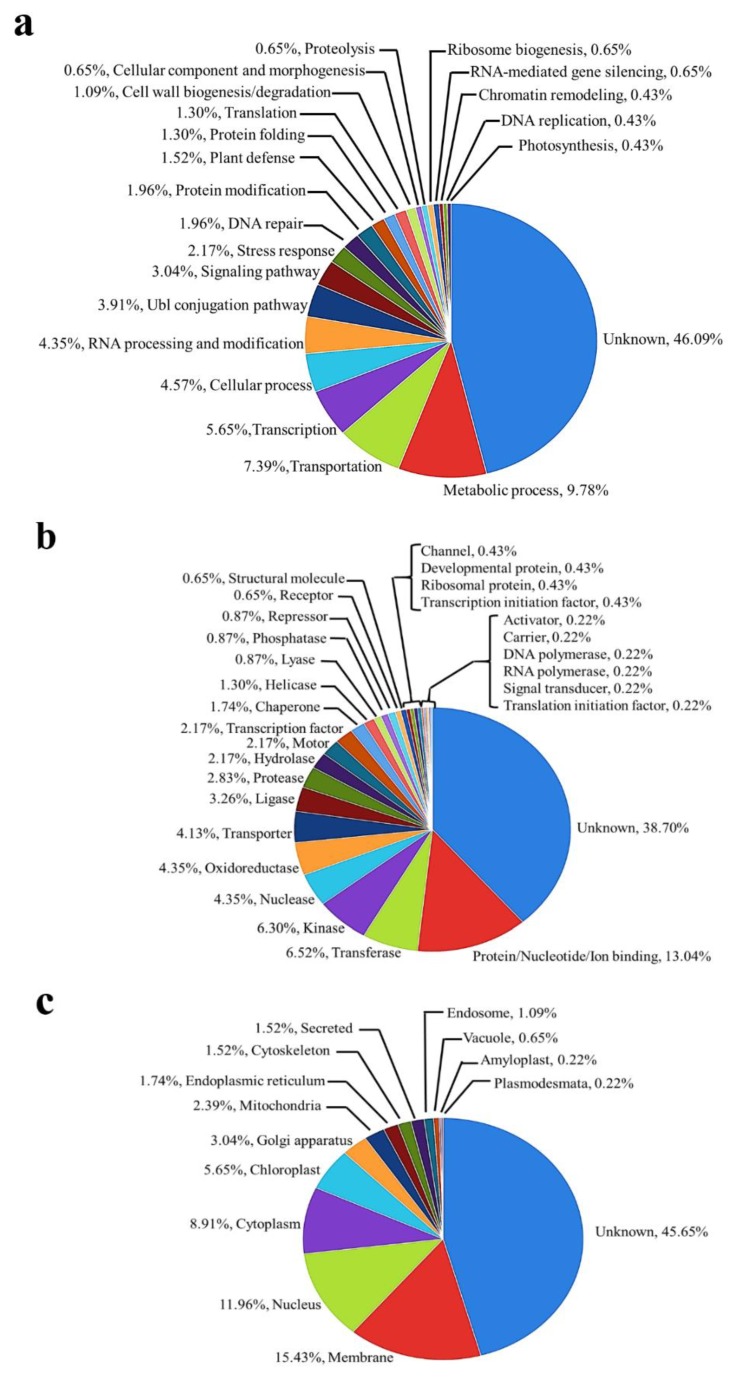
Biological function and subcellular localization of 460 identified phosphoproteins during somatic acquisition and plant regeneration. (**a**) Assignment of the 460 identified phosphoproteins to biological process categories with the use of gene ontology (GO) classification. (**b**) Assignment of the 460 identified phosphoproteins to molecular function categories with the use of GO classification. (**c**) Assignment of the 460 identified phosphoproteins to subcellular localization categories with the use of GO classification.

To classify the identified underlying phosphoproteins based on gene ontology (GO) classification tools, the total identified phosphoproteins were categorized into groups based on their percentage of presence in biological processes, from the highest amount, as follows: unknown (46.09%), metabolic process (9.78%), transportation (7.39%), and transcription (5.65%) (Figure 3a). The molecular functions of the identified phosphoproteins were described as percentages as follows: unknown (38.70%), protein/nucleotide/ion binding (13.04%), transferase (6.52%), and kinase (6.30%) (Figure 3b). The cellular components of the identified phosphoproteins presented the highest percentages as follows: unknown (45.65%), membrane (15.43%), nucleus (11.96%), and cytoplasm (8.91%) (Figure 3c). 

### 2.3. Differentially Expressed Phosphoproteins during Acquisition of Oil Palm Somatic Embryo 

Expressed proteins from embryogenic callus acquisition to somatic embryo and somatic embryo differentiation were examined. Two proteins were specifically expressed during somatic embryo differentiation: the E3 ubiquitin-protein ligase At1g63170-like isoform X2 and sister chromatid cohesion protein PDS5 homolog A isoform X2. Ten proteins were specifically expressed during embryogenic callus acquisition for the somatic embryo and plantlet but were suppressed in the cotyledonary stage, namely uncharacterized protein LOC105047683 isoform X3, pentatricopeptide repeat-containing protein At4g39530, zinc-finger homeodomain protein 6-like, uncharacterized protein LOC105048836 isoform X1, endoribonuclease Dicer homolog 1 isoform X1, cationic amino acid transporter 1-like, AT-rich interactive domain-containing protein 4-like, DNA polymerase eta isoform X1, probable UMP/CMP kinase 4, and uncharacterized protein LOC105047412. Pollen-specific leucine-rich repeat extensin-like protein 2 was specifically expressed in the callus, globular, torpedo and cotyledonary stages but suppressed in the plantlet stage. Probable inactive receptor kinase At1g48480 was specifically expressed in the callus, globular and torpedo stages but suppressed in the cotyledon and plantlet stages (Table 1). To characterize the phosphorylation sites from differentially expressed phosphoproteins, phosphorylation sites were predicted by the NetPhos 3.1 sever. The results showed that 14 candidate proteins have a phosphorylation site either at serine, threonine or tyrosine residues (Table 1).

### 2.4. Quantitative Real-Time Reverse Transcription–PCR of Differential Phosphoproteins

**Figure 4:**
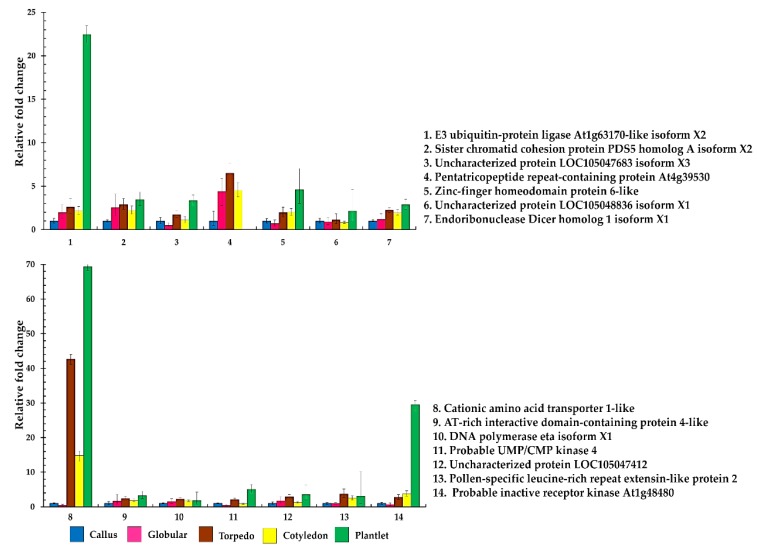
mRNA expression patterns derived from differentially expressed phosphoproteins.

To verify differentially expressed proteins during embryogenic callus acquisition for the somatic embryo and somatic embryo differentiation, the quantitative real-time reverse transcription–PCR technique was used. We analyzed the mRNA expression level of 14 differentially expressed genes. For all genes, the fold changes of the transcript level were calculated by using the expression level at the callus stage as a reference. The result showed that genes were expressed in all stages except pentatricopeptide repeat-containing protein At4g39530, which was suppressed in the plantlet stage. The expression of 14 genes showed a similar pattern of up-regulation during somatic embryogenesis, which partially correlated well with the protein intensity from the nano LC-MS/MS result (Figure 4).

## 3. Discussion

Plants have special characteristics, known as their totipotency abilities, that cells can reprogram to act as stem cells. After the cells reprogram themselves, they can multiply and develop into a completely new plant. Somatic embryogenesis is a powerful process that underpins the biological change to the acquisition of totipotency cell capacity. Previous studies have shown that phosphoproteins are involved in plant differentiation and development. Many phosphorylated proteins can be modified by different kinds of protein kinases that regulate multiple processes, including the biological metabolism and the hormone response [15]. In this study, we collected samples during the embryogenic callus, somatic embryo maturation (globular, torpedo and cotyledonary stages), and plantlet stages of oil palm tissues to identify and analyze differentially expressed, post-translational phosphoproteins. Four hundred and sixty proteins were identified from LC-MS/MS protein analysis (Supplementary data Table S1). Many identified phosphoproteins were found in all stages, which is similar to previously reported phosphoproteins which were found in the early stages of rice embryo germination [19]. They are involved in carbohydrate metabolism and protein synthesis/degradation: these proteins include starch synthase 3, chloroplastic/amyloplastic, cellulose synthase-like protein D1, fructokinase-6, chloroplastic, and the E3 ubiquitin-protein ligase At1g63170-like isoform X2 [19]. Our results revealed several phosphoproteins that were previously identified during seed development in *Arabidopsis*, rapeseed, and soybean, such as GATA transcription factor 26 isoform X2, acyl-CoA-binding domain-containing protein 5-like isoform X1, and mitogen-activated protein kinase YodA-like [20], indicating that the phosphorylated proteins play important roles in the biological mechanism during embryo germination and seed development. Moreover, previously identified proteins that are involved in somatic embryogenesis such as transcription factor bHLH68-like, transcription factor bHLH113-like, ethylene-responsive transcription factor LEP-like, and somatic embryogenesis receptor kinase 2-like [21] were also found in this study (Table S1).

All identified phosphoproteins were categorized into different biological processes, molecular functions, and cellular components based on the gene ontology (GO) classification system. In the biological process, the majority of identified proteins were characterized in the metabolic process, transportation and transcription. When classified based on molecular function, the highest percentage of proteins was characterized in protein, nucleotide, and ion binding, transferase, and kinase, respectively. For the cellular component, the identified proteins showed the highest percentages as the membrane, the nucleus and the cytoplasm, respectively. This result showed a similar pattern to the gene ontology result of phosphoproteome characterization during rice embryo in the early stages of germination [19,22], which showed a highly classified function as in metabolic processes and was also similar to the gene ontology result of the phosphoproteomic function analysis of seed maturation in *Arabidopsis*, rapeseed, and soybean [20]. Moreover, our study identified phosphoproteins that were also found during the developmental reprogramming of the moss *Physcomitrella patens* [17]. This information indicated that the identified phosphoproteins may have proteins with an important role in the control and regulation of cellular metabolism by inducing gene expression and gene-related kinase cascades to induce cellular development and morphological changes during oil palm tissue culture.

The abundance of differential phosphoproteins during somatic embryo maturation and regeneration has been demonstrated (Table 1). The E3 ubiquitin-protein ligase At1g63170-like isoform X2 was expressed only in somatic embryo maturation except in the embryogenic callus; this protein is involved in the regulation of biological processes, including the hormonal control of vegetative growth, plant reproduction, light response, biotic and abiotic stress tolerance, and DNA repair; this indicates a major role for protein degradation in the control of plant life [23]. In a previous study, E3 ubiquitin ligases were found to comprise a ubiquitin-mediated proteasome cascade pathway that acts as a central regulator in hormone signaling in plants by controlling protein stability [24]. E3 ubiquitin ligases facilitate the transfer of ubiquitin moieties to substrate proteins marked for degradation that serve as central regulators in many cellular and physiological processes in plants [25]. Moreover, the probable E3 ubiquitin-protein ligase ARI1 has been reported to be involved in embryo development and pathways such as controlled proteolysis in conifers. The ubiquitin protein ligase plays a major role as a robust marker of correct embryo development in *Pinus pinaste* [26] and the somatic embryogenic lines of *Pseudotsuga menziesii* [Mirb.] [27]. In our study, the sister chromatid cohesion protein PDS5 homolog A isoform X2 was specifically expressed in the globular, torpedo and plantlet stages that regulate chromosome segregation during mitosis and meiosis and was also found to play a role in DNA repair. The depletion of AtPDS5 proteins has a weak impact on meiosis but leads to severe effects on development, fertility, somatic homologous recombination (HR), and DNA repair [28]. In our study, ten proteins were expressed only in the embryogenic callus acquisition, somatic embryo, and plantlet stages. We identified seven characterized proteins and three uncharacterized proteins. The pentatricopeptide repeat-containing protein functions in both the mitochondrion and the nucleus. In the mitochondrion, it is associated with polysome and may play a role in the translation required during embryogenesis. In the nucleus, it might be involved in the regulation of its own gene expression [29]. Zinc-finger homeodomain protein 6-like is a transcription factor that plays a regulatory role in *Arabidopsis* floral development [30] and may be involved in wheat inflorescence development, biotic pathways, and abiotic signaling pathways [11]. The endoribonuclease Dicer homolog 1 is involved in RNA-mediated post-transcriptional gene silencing (PTGS) in the microRNAs (miRNAs) biogenesis pathway. In a previous report, endoribonuclease Dicer homolog 1 acted as DCL1 and DCL3, which promote flowering via the repression of the *FLOWERING LOCUS C* (*FLC*) gene [31]. Cationic amino acid transporter 1-like protein functions as a proton symporter of basic amino acid transporters at the plasma membrane. The amino acid uptake plays many other metabolic and signaling roles in plants. The AT-rich interactive domain containing protein 4-like acts as a DNA binding protein and directly binds to the AT rich promoter element in the auxin biosynthetic gene. It plays a major role in shoot apical meristem (SAM) development in rice [32]. DNA polymerase beta is involved in DNA synthesis during DNA replication, increasing resistance against ultraviolet light [33,34]. The UMP-CMP kinase is involved in the pyrimidine biosynthetic pathway and is elevated during seedling development and fruit ontogeny [35]. Here, pollen-specific leucine-rich repeat extensin-like protein 2 was specifically expressed in the callus, globular, torpedo and cotyledonary stages and was suppressed in the plantlet stage. Pollen-specific leucine-rich repeat extensin-like protein 2 is involved in modulating cell morphogenesis by regulating cell wall formation and assembly, growth polarization, and pollen tube growth [36]. Here, probable inactive receptor kinase At1g48480 was specifically expressed only in the callus, globular and torpedo stages and was involved in the activation of the YodA MAP kinase cascade, regulating the asymmetric first division and embryo polarity programming [37]. 

To confirm the mRNA transcription level of identified phosphoproteins, the candidate genes were verified by using quantitative real-time PCR. The transcription level of 14 genes was expressed in all stages except for pentatricopeptide repeat-containing protein At4g39530, which was suppressed in the plantlet stage (Figure 3). These results showed similar patterns to a previous study, which identified genes associated with oil palm somatic embryogenesis when using cDNA amplified fragment length polymorphism (AFLP) [38]. However, the mRNA and protein abundances did not always correlate. Often, we observed the expression at the mRNA level but not at the protein level, because of the different regulation at transcription and translational steps [39]. This result indicates that the mRNA transcription level in the callus and globular stages may undergo dedifferentiation and redifferentiation responses in a different way to the torpedo and cotyledon stages, which may need to prepare itself for the specific tissue developmental process and metabolic process response.

In conclusion, we identified phosphoproteins during the embryogenic callus transition to the somatic embryo that are involved in cellular differentiation, cell morphogenesis, embryo polarity programming, and signaling roles in oil palm. The phosphoproteins identified in this study serve as good candidates for the development of protein biomarkers that are specific to somatic embryogenesis and can be used to understand the mechanism of the somatic embryogenesis process. Further studies are needed to characterize the function of candidate phosphoproteins for understanding somatic embryogenesis process in vivo during oil palm tissue culture.

## 4. Materials and Methods

### 4.1. Plant Materials

Oil palm cv. Tenera zygotic embryos at 15 weeks after pollination, taken from the Rice Science Unit, Kasetsart University, Kamphaengsaen campus, Thailand, were used as explants for tissue culture. The zygotic embryo explants were cultured on an N6 medium pH 5.75 containing 2 mg L^−1^ of 2,4-D, 30 g L^−1^ of sucrose, and 2 g L^−1^ of phytagel for callus induction under dark conditions; these embryos were subcultured every month for 3–5 months until the polyembryoids (globular stage) differentiated [5]. The embryogenic callus was transferred to an N6 medium supplemented with 0.1 mg L^−1^ of 2,4-D, 0.16 g L^−1^ of putrescine, 0.5 g L^−1^ of casein amino acids, 30 g L^−1^ of sucrose, 2 g L^−1^ of phytagel, and 2 g L^−1^ of activated charcoal under a cool-white fluorescent lamp (50–60 lmol m^−2^ s^−1^) for 16 h photoperiod until the somatic embryo stages at the globular, torpedo and cotyledon stages were found [5]. The small shoot was then transferred to a modified N6 medium containing 0.5 g L^−1^ activated charcoal and 30 g L^−1^ sucrose under photoperiod light condition for 16 h to regenerate a completely new plantlet [5]. Oil palm tissue samples at the embryogenic callus stage and somatic embryo stages (globular, torpedo and cotyledonary), as well as regenerated plantlets, were collected for protein extraction (Figure 1).

### 4.2. Protein Extraction and Quantification

The protein extraction protocol was performed according to the methodology of Kaweewong et al. [40]. Plant samples were ground to pulverization in liquid nitrogen. A total of 0.5 g of pulverized samples was then dissolved in 0.5% SDS with a continuous vortex for 30 min at room temperature. The pellet was precipitated by centrifugation at 12,000 rpm for 15 min at 4 °C. The supernatant was transferred to a new microcentrifuge tube, mixed well by an equal volume of cooled acetone, and incubated for 2 h at −20 °C. The mixture was then centrifuged at 12,000 rpm for 15 min at 4 °C to collect pellet materials. The pellet was washed by cooled acetone and centrifuged at 12,000 rpm for 15 min at 4 °C, three times. The pellet was dissolved again in 0.5% SDS and desalted by a ZebaTM Desalt spin column (ThermoFisher Scientific, Waltham, MA, USA) according to the manufacturer’s instructions. The concentration of soluble protein was determined by using the Lowry method [41]. The protein was stored at −80 °C until use in the phosphoprotein enrichment experiment.

### 4.3. Phosphoprotein Enrichment and Digestion

Phosphoproteins were analyzed according to the phosphoproteome analysis workflow method [42,43]. Each 500 µg protein sample was used for phosphoprotein enrichment by using the Pierce^®^ Phosphoprotein Enrichment Kit and desalted by the ZebaTM Desalt spin column (Thermo Fisher Scientific, Waltham, MA, USA), according to the manufacturer’s protocol. To reduce disulfide bonds, 10 mM dithiothreitol (DTT) in 10 mM ammonium bicarbonate was added to one microgram of purified phosphoproteins while the reformation of disulfide bond in phosphoproteins was blocked by alkylation with 30 mM iodoacetamide (IAA) in 10 mM ammonium bicarbonate. The phosphoprotein samples were digested with 400 ng of sequencing grade porcine trypsin (Promega, Walldorf, Germany) for 16 h at 37 °C. The tryptic peptides were dried by using a speed vacuum concentrator and resuspended in 0.1% formic acid (FA) for nano LC-MS/MS analysis. 

### 4.4. Nano-Liquid Chromatography–Mass Spectrophotometry (Nano LC-MS/MS)

Phosphopeptide samples were injected in triplicate into the Impact II UHR-TOF MS system (Bruker Daltonics Ltd., Germany) coupled to a nano LC system, the UltiMate 3000 LC System (Thermo Fisher Scientific, Waltham, MA, USA), by using an electrospray at the flow rate of 300 nL/min with a nanocolumn (PepSwift monolithic column 100 µm i.d. × 50 mm). Mobile phases of solvent A (0.1% formic acid) and solvent B (80% acetonitrile and 0.1% formic acid) were used to elute peptides with a linear gradient from 10%–70% of solvent A at 0–13 min (the time-point of retention time), 90% solvent B at 13–15 min to remove all peptides in the column, and a final elution of 10% B at 0–15 min to remove the remaining salt. The resolution of the MS step is 0.6 and the accuracy was 0.15 u (m/z). 

### 4.5. Bioinformatics and Data Analysis

The DeCyder MS 2.0 analysis software (GE Health-care, Chicago, IL, USA) was used to measure the protein intensity based on peptide MS signal intensities of the individual LC-MS analyzed data. To evaluate the peptide from each stage, the PepDetect module was used to produce ion peptides with the following data set: mass resolution, 0.6; typical peak width, 0.5; time of flight (TOF) resolution, 10,000; charge status, from 1 to 10; and m/z shift tolerance, 0.1 u. The PepMatch module evaluated the signal intensity maps from each sample. The highest-intensity sample was used as a control, presenting the relative abundance of peptides as 2log intensities with mass tolerance set to 0.5 amu. An average abundance ratio of more than two-fold was determined as an over-expressed protein with a significant standard *t*-test and one-way ANOVA *p* < 0.05. All MS/MS spectra from the Decyder MS analysis were performed by applying the global variable mode of carbamidomethyl, variable mode of oxidation (M), phospho (ST) and phospho (Y), peptide charge state (1+, 2+ and 3+), and m/z tolerance 0.1 u; these spectra were searched against NCBI protein databases (https://www.ncbi.nlm.nih.gov/) with an *Elaeis guineensis* genome (39,705 sequences; 4 February 2017) to identify matching peptides by using the Mascot software search engine tool (Matrix Science, London, UK). Identified proteins were filtered with a one-way ANOVA *p* < 0.05. In this experiment, 1 g of BSA was used as an internal standard to normalize protein intensities from each set of data. Protein function classification, annotations, and subcellular location were identified by using gene ontology (GO), Uniprot (http://www.uniprot.org/), PANTHER (http://pantherdb.org/), and search tools. Matching proteins from each sample were used to determine the similarity and differential protein expression by using the Bioinformatics and Evolutionary Genomics tool (http://bioinformatics.psb.ugent.be/webtools/Venn/) for collect candidate proteins to verify differential mRNA expression level by using quantitative real-time reverse transcription–PCR. The identified phosphoproteins were used to predict the phosphorylation by using their peptide sequences with NetPhos 3.1 sever (http://www.cbs.dtu.dk/services/NetPhos/). The program identified specific residues—p-Thr, p-Ser and p-Tyr—for phosphorylation sites. 

### 4.6. Quantitative Real-Time Reverse Transcription–PCR Verification

The total RNA was extracted from the primary callus and somatic embryo at the globular, torpedo and cotyledon stages, as well as from plantlets, by using the Spin Plant RNA kit (STRATEC Molecular, Germany) according to the manufacturer’s protocol with minor modifications by treatment with DNAase I (Vivantis, Selangor, Malaysia) on the RNA binding column before eluting the total RNA. One microgram of total RNA was used to synthesize the first strand cDNA by using a cDNA synthesis kit (Biotechrabbit GmbH, Berlin, Germany) according to the manufacturer’s instructions. The cDNA content of *E. guineensis* elongation factor alpha-1 was used as a normalization of the expression level. The expression level of the candidate proteins was examined with designed specific primers (Supplementary data Table S2) by using the KAPA SYBR^®^ FAST qPCR kit Mastermix (2x) universal (kapa biosystems, Wilmington, MA, USA) according to the manufacturer’s instructions on an Eppendorf Mastercycler ep Realplex (Thermo Fisher Scientific, Waltham, MA, USA). The amplification for each gene was assessed for three replications, and the transcription level of each gene was confirmed in three bioreplications. The double delta Ct calculation was used to determine the fold change by comparison with the mRNA level of the callus stage as a calibrator.

## Figures and Tables

**Table 1:** List of differentially expressed phosphoproteins.

NCBI Accession Number	Protein Name	Peptide Sequence and Predicted Phosphorylation Site (*)	MOWSE Scores	Log_2_ Abundance				
				Callus	Globular	Torpedo	Cotyledon	Plantlet
gi|743754692	E3 Ubiquitin-Protein Ligase	GAET *NS *ENICNGGTVAS *GT *DR	12.71	0	16.51	15.48	16.50	17.08
gi|743807360	sister chromatid cohesion protein PDS5	KNS *MKKS *S *S *S *K	8.51	0	16.01	14.62	0	17.66
gi|743797598	uncharacterized protein	LVLRS *LR	1.36	15.42	15.05	12.95	0	16.23
gi|743821075	pentatricopeptide repeat-containing protein	MPERNMISWSS*MISMY *TQHNR	10.70	15.09	15.66	14.32	0	17.13
gi|743851198	zinc-finger homeodomain protein 6-like	GSNS *T *GGGAGGGPMT *ES *S *S *EER	16.71	15.33	15.42	14.49	0	18.64
gi|743802384	uncharacterized protein	RAWMPTGCLK	8.43	15.72	16.48	15.18	0	18.13
gi|743828992	endoribonuclease Dicer	GVS *Y *CK	4.14	15.97	16.55	15.26	0	19.01
gi|743856309	cationic amino acid transporter 1-like	RGAT *GVAS *AEK	9.96	14.42	17.73	15.59	0	18.29
gi|743774060	AT-rich interactive domain-containing protein 4-like	T *DGLEY *ICPHCSLS *NYK	6.62	15.72	16.83	15.98	0	17.38
gi|743882271	DNA polymerase beta X1	IQEDAMKLFVSGLR	12.10	16.93	18.02	16.36	0	18.83
gi|743855052	UMP/CMP kinase 4	MVDAS *KDVDGSFPGEK	5.19	16.20	17.54	16.80	0	19.48
gi|743796037	uncharacterized protein	DAPFPVTTS *FYFHAKSLDPDR	13.44	18.02	17.81	18.19	0	17.66
gi|743880173	pollen-specific leucine-rich repeat extensin-like protein	GS *S *AMKSS *PLAS *S *AAS *S *GGSTNY *T *R	7.82	15.40	15.54	13.69	15.75	0
gi|743771831	inactive receptor kinase	VAGY *RAPEVTDARKVS *QK	9.61	15.84	16.27	14.72	0	0

## Supplementary Materials

The following are available online at https://www.mdpi.com/2223-7747/9/1/36/s1, Table S1: Phosphoprotein sequence matched to proteins in the FTP site *Elaeis guineensis* database (39,705 sequences), Table S2: Primer pairs used in quantitative real-time reverse transcription–PCR.
